# Behavioral Biometric Data Analysis for Gender Classification Using Feature Fusion and Machine Learning

**DOI:** 10.3389/frobt.2021.685966

**Published:** 2021-05-07

**Authors:** Shivanand S. Gornale, Sathish Kumar, Abhijit Patil, Prakash S. Hiremath

**Affiliations:** ^1^ Department of Computer Science, Rani Channamma University, Belagavi, India; ^2^ Department of MCA, KLE Technological University, Hubballi, India

**Keywords:** biometrics, gender classification, offline handwritten signature, LBP, HOG, K-NN, decision tree, support vector machine

## Abstract

Biometric security applications have been employed for providing a higher security in several access control systems during the past few years. The handwritten signature is the most widely accepted behavioral biometric trait for authenticating the documents like letters, contracts, wills, MOU’s, etc. for validation in day to day life. In this paper, a novel algorithm to detect gender of individuals based on the image of their handwritten signatures is proposed. The proposed work is based on the fusion of textural and statistical features extracted from the signature images. The LBP and HOG features represent the texture. The writer’s gender classification is carried out using machine learning techniques. The proposed technique is evaluated on own dataset of 4,790 signatures and realized an encouraging accuracy of 96.17, 98.72 and 100% for k-NN, decision tree and Support Vector Machine classifiers, respectively. The proposed method is expected to be useful in design of efficient computer vision tools for authentication and forensic investigation of documents with handwritten signatures.

## Introduction

Now days, Biometrics is a widely established measure in security and authentication processes in several system applications. According to International Standard Organization (ISO), biometric is defined as a means of biological process for recognizing and analyzing an individual based on their physiological (fingerprint, face, iris, palm prints) and behavioral characteristics (Signature, Gaits, keystroke, voice/speech) (Fairhurst et al., 2017). Among these traits, handwritten signature is the most preferred age-old behavioral trait, which is used to authenticate the documents due to its individuality and consistence features. Signatures were first knowingly recorded in the Talmud (Steinsalz, 1992) in the IV century. Since then the nature of handwriting signature has been continuously evolving, in the past decades, from traditional way of handwritten signature with pen or pencil on a piece of paper to digitally recording signature, i.e. on a tablet by digitizing pen. Nowadays, with the advent of the electronic scanning devices, handwritten signatures are digitized and then stored for use in automatic document verification and authentication processes. Signatures basically comprise horizontal, vertical and curving strokes with multilingual texts which mostly denote name and last name, as the description made by its handler/person, representing his agreement or presence on that document. Handwritten signature is significant as a handy tool for identifying and verifying the document as well as for authorizing the user of a document. Even today, signatures are still considered as the most reliable and adequate evidences in the court of law. Signature, being a behavioral biometric trait, measures the identity of an individual uniquely. The handwritten-signature-trait contains many dynamic and inherent behavioral features, which are useful in the determination of a person’s soft characteristics like gender, handedness, personality and age information, etc. (Jain et al., 2004) (Singh and Verma, 2019).

In this paper, the objective of the proposed work is to design a machine learning framework for analyzing the gender classification of writers based on their handwritten signatures using fusion of textural and statistical features. The LBP and HOG features represent the texture. The k-NN, decision tree and SVM classifiers are used to perform classification. Further, the paper is organized as follows. *Related Work* contains the literature on the work related to the signatures based gender classification. *Proposed Methodology* focuses on the proposed methodology. In *Experimental Results and Analysis*, experimental results are analyzed and compared with the existing results. Finally, in *Conclusion*, the conclusions are given.

## Related Work

In the literature on the recognition of handwritten signature as a biometric trait, a considerable number of studies have been reported on specific applications such as signature verification, handwriting recognition and writer identification. However, work related to writer’s gender classification domain is very scanty. In this section, a review of related studies on gender classification is presented.

A. A. M. Abushariah et al. (Abushariah et al., 2012) have performed signature based gender classification using the global features, namely, height, width and area, on own image dataset of 3,000 signatures of 50 males and 50 females. By using artificial neural network (ANN), an average accuracy of 76.20 and 74.20% for male and female, respectively, has been obtained.

Tasmina Islam et al. (Islam and Fairhurst, 2019) have investigated the effect of writer style, age, and gender on Natural Revocability Analysis in Handwritten Signature Biometrics. A dataset of a total of 4,190 signatures were collected from different gender and age groups, and sixty features were extracted. Signatures were analyzed using the Analysis of Variance (ANOVA) technique and it is found that adopting different style signatures will not affect the new naturally revoked signature. There is no much difference in the static and dynamic features of original and new signatures. Natural revocability can be the feasible option in demographic factors such as age and gender of the handwritten signatures.

Prasentjit Maji et al. (Maji et al., 2015) have performed gender classification based on offline handwritten signature by using basic statistical features over a dataset of 500 signatures. By using back propagation neural network, an accuracy of 84%has been achieved.

Cavalcante Bandeira, et al. (Cavalcante Bandeira et al., 2019) have investigated the impact of the combination of a handwritten signature and keyboard keystroke dynamics for gender prediction. From 100 participants, keystroke characteristics and handwritten signature images were collected. By extracting basic static statistical and dynamic features, an average accuracy of 68.03% is obtained with multilayer perceptron.

Moumita Pal, et al. (pal et al., 2018) have performed gender classification using Euler number based feature extraction on 500 Hindi handwritten signatures images. Using back propagation neural network classifier, an average accuracy of 88.80% is obtained.

Many researchers have worked on writer identification of handwritten signatures and handwritings (Al-ma'adeed and Hassaïne, 2014; Ibrahim et al., 2014; Siddiqi et al., 2014; SRIVASTAVA, 2014; Mirza et al., 2016; Bouadjenek et al., 2017; Topaloglu and Ekmekci, 2017; Navya et al., 2018; Baboria et al., 2019; Faundez-Zanuy et al., 2020). However, it is observed that very less effort is done on gender identification based on offline handwritten signatures. These signatures may contain multilingual texts. Therefore, to bridge this research gap, a frame work is proposed to identify writer’s gender from handwritten signatures of individuals with varying ages.

## Proposed Methodology

**FIGURE 1:**
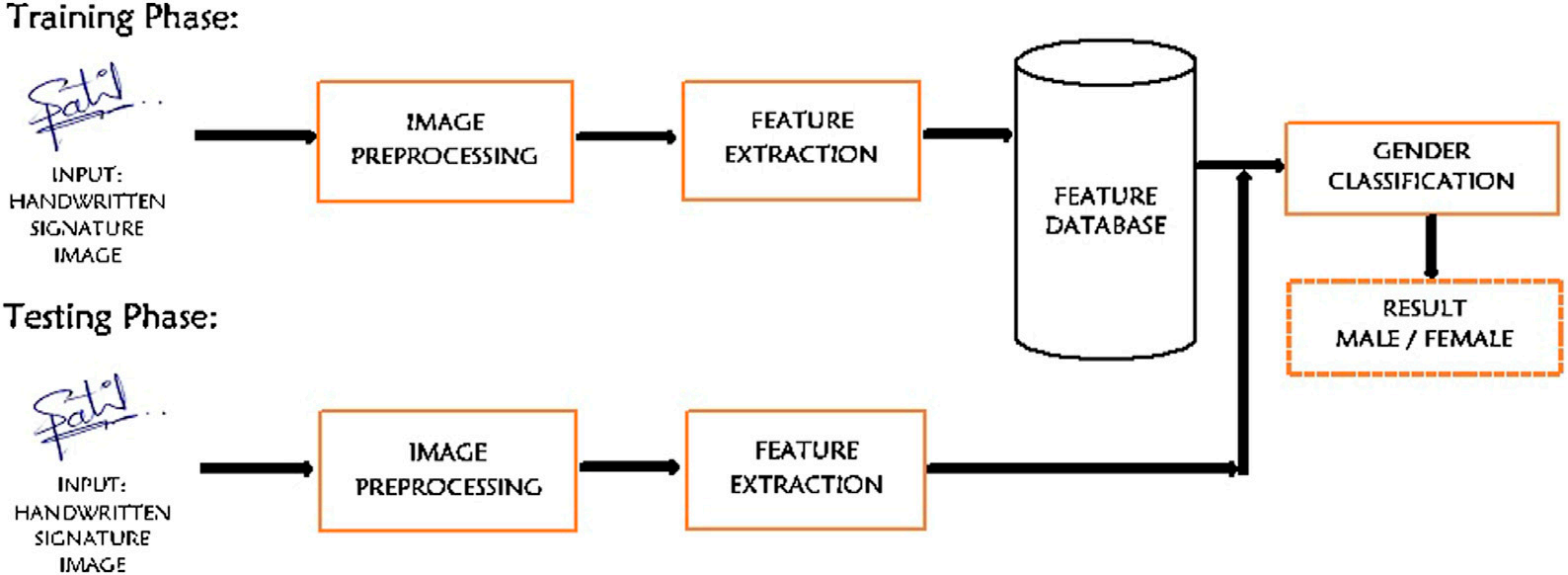
General methodology for the gender classification using handwritten signature.

The general steps involved in the proposed writer’s gender classification based on handwritten signature biometric are shown in Figure 1.

### Dataset

From the literature review, it has been found that the publicly available standard image datasets of handwritten signatures do not have gender annotation. With this motivation, own dataset is created to carry out the experiments with the proposed method. The nature of this own offline handwritten signature database is described below.

• The signature samples are collected from persons with varying ages. These samples consist of multilingual scripts of Kannada, Hindi, Marathi, and English.
• Each individual has done 10 signatures on a white A4 paper sheet using blue or black colored ball pen.
• To avoid geometrical variations further, the papers with sample signatures have been scanned using the EPSON DS-1630 color scanner with a resolution of 300 DPI.
• The database consists of 4,790 signatures, which are acquired from 479 subjects, out of whom 250 are male volunteers and 229 are female volunteers.
• All the individuals, who had knowledge of English and other languages, have been educated about the purpose of collection of signature samples.

**FIGURE 2:**
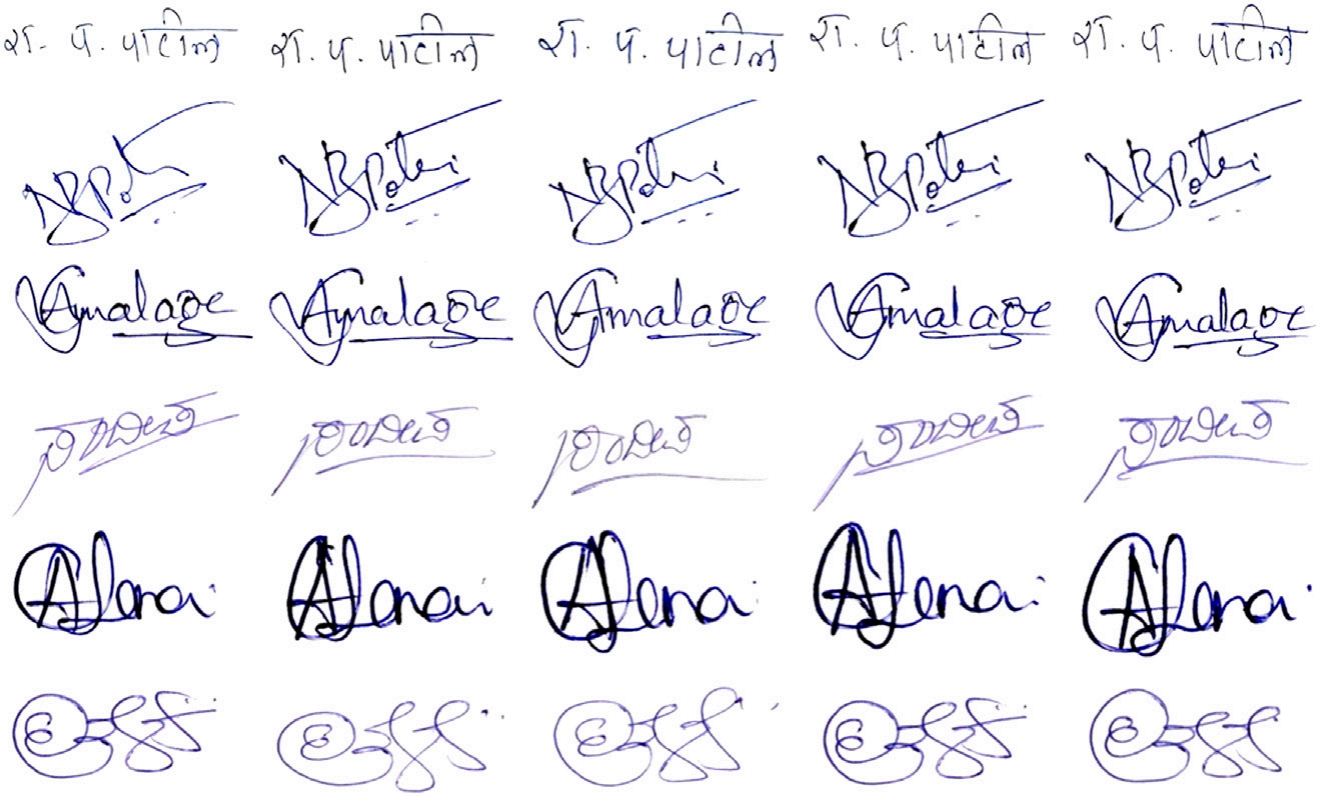
Handwritten signature samples from the own dataset.

Some samples of these signatures are shown in Figure 2.

### Preprocessing

**FIGURE 3:**
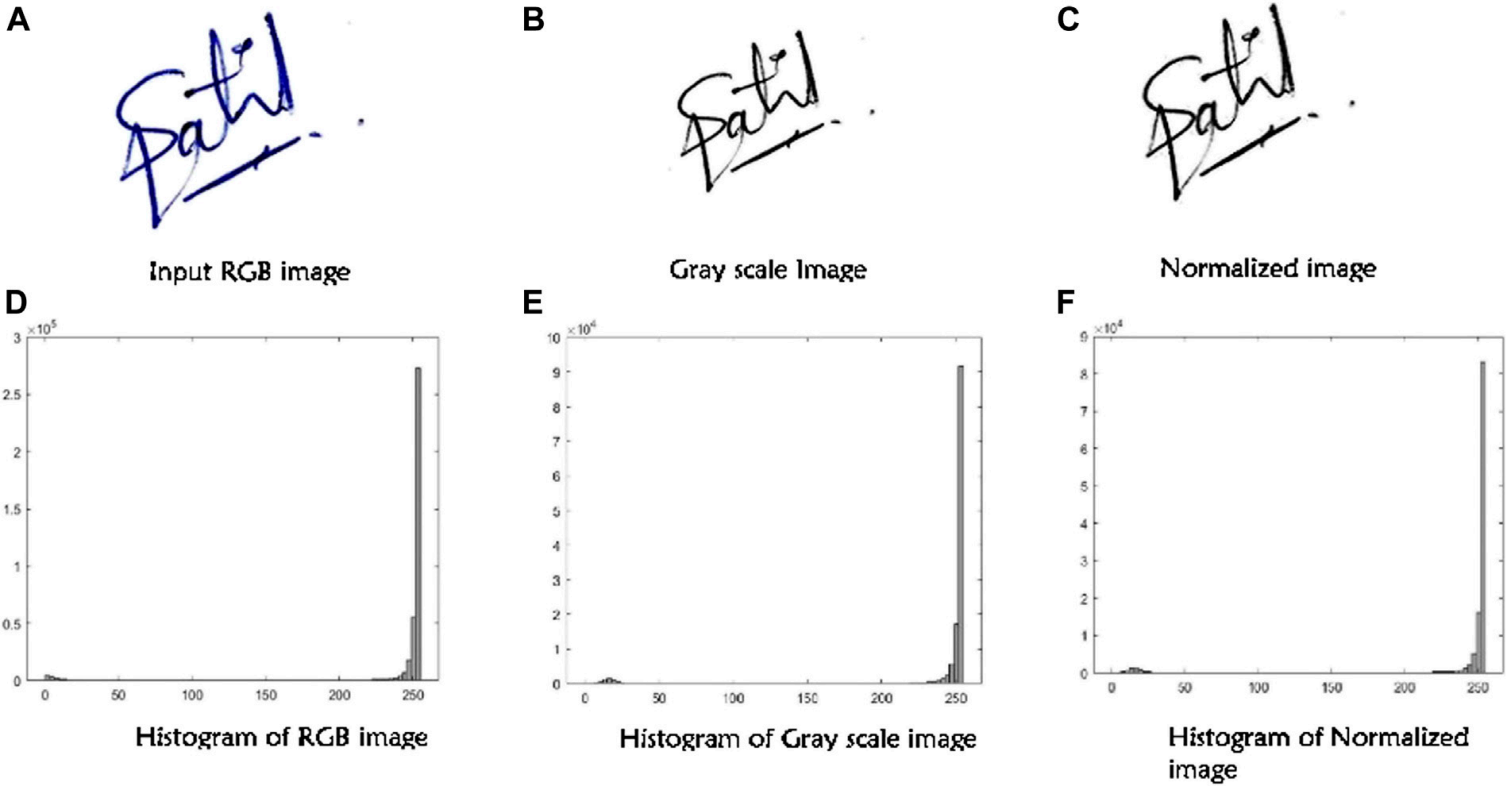
Preprocessing of signatures.

The main aim of pre-processing is to improve the data and remove unwanted distortion or noise and also to enhance important features of handwritten signatures. The various techniques are taken into consideration such as converting the raw handwritten signature input image to a standard Grayscale, than normalizing images by cropping into a standard size of 150 × 150. As shown in the Figure 3.

### Feature Extraction

Feature extraction for object recognition in the image is the most important part of the gender classification approach (Gornale et al., 2020). In this proposed work, three different feature extraction approaches (the first one is the HOG approach and the second one is the LBP approach and the third one is the Statistical approach) and Textural features are used. The details of these approaches are given below.

#### Local Binary Patterns

**FIGURE 4:**
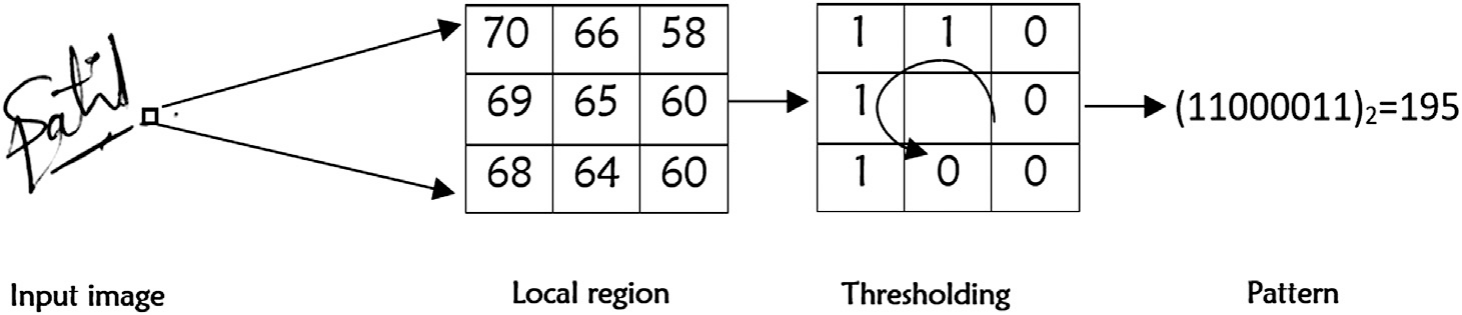
LBP computation.

LBP was first introduced by Ojala et al., in 1996, as an efficient and powerful texture descriptor, which is widely used in image processing and computer vision areas for feature and histogram representation (Ojala et al., 1996). The original LBP operator considers eight neighborhood of a pixel, with Center pixel being used as threshold (Shivanand, 2017). It generates an 8-bit binary pattern for each pixel depending on its 3 × 3 neighborhood, which is converted to the corresponding decimal value, as illustrated in Figure 4. The distribution of these binary patterns, represented by the equivalent decimal, serve as the local texture descriptor. In general, for a pixel at i_c_ = (x_c_, y_c_), the LBP is calculated by using the following formula:

$$\text{LBP }\left(x_{c},y_{c}\right)=\sum_{n=0}^{N-1}s\left(i_{n}-i_{c}\right)2^{n},$$ (1)

where i_n_ is a neighbor pixel of the pixel at i_c_ and s = 1 if i_n_ ≥ i_c_ otherwise s = 0. From each signature image, 58 LBP features are extracted (Shivanand, 2017).

#### Histogram of Oriented Gradients

The HOG, first introduced by Dalal and Triggs (Dalal and Triggs, 2005), is one of the efficient local texture descriptor that is often utilized in the computer vision and image processing for capturing the distribution of the edges of an object or its local intensity gradients value (R et al., 2019). The HOG feature vector is calculated from the image gradient detectors, each convolved with a simple convolution window as follows:

$$\begin{array}{l} Gx=I\left(x+1,y\right)-I\left(x-1,y\right),\\ Gy=I\left(x,y+1\right)-I\left(x,y-1\right), \end{array}$$ (2)

where, I (x, y) is the pixel intensity at location (x, y) in the input image; Gx and Gy are the horizontal and vertical components of the gradients, respectively.

#### Statistical Features

Statistical features are used to characterize the location and variability of the pixel distribution in a signature image. In this work, the statistical features and shape features of handwritten signatures that are computed are as following:

• Area is the number of pixels in an image.
• $\text{Skewness}=\frac{E{\left(x-\mu \right)}^{2}}{\sigma^{3}}.$
• Euler Number = (number of objects)−(number of holes).
$\text{Euler}\left(n\right)=2^{n}\left(n,\frac{1}{2}\right).$
• Perimeter is the number of boundary pixels.
Perimeter = bwperim(BW)
• Eccentricity is ratio of the distance between the foci of the ellipse and major axis length. $\text{Eccentricity}\;e=\left(\frac{1-b^{2}}{a^{2}}\right)\cdot \frac{1}{2}.$
• EquivDiameter is the diameter of the circle with same area as region. $\text{Equiv}-\text{diameter}\;d=\sqrt[{2}]{\frac{\text{Area}}{\pi }}.$
• Standard Deviation is one of the quantitative measurement. $\text{Standard}\;\text{Deviation}=\sqrt{\frac{1}{N-1}}\sum_{i=1}^{N}{\left|Ai-\mu \right|}^{2}.$

#### Textural Features

• $\text{Entropy}\;\text{filter:M}=\text{Entropy}\left(\text{H}\right)=\sum_{i=1}^{n}p_{i}\left(\log_{2}p_{i}\right),$
• Standard filter S = stdfilt(f),
• Range filter J = rangefilt(I).

All the features determine the center element of the neighborhood (Gornale et al., 2016).

#### Feature Level Fusion

The feature level fusion is sparingly utilized due to its complexity of larger dimensions in fused feature space (Gornale et al., 2016). However, the handwritten signature have inherently rich sets of characteristics and thus, intuitively, fusion of features is expected to be more effective. Thus, in this work, the features of handwritten signatures obtained from HOG, statistical and LBP techniques have been fused together by using concatenation rule (Gornale et al., 2019).

### Classification

K-Nearest Neighbor (k-NN) Classifier: k-NN classifier classifies the data based upon the different kinds of distances selected for the purpose (Gornale et al., 2016). It classifies the object feature vectors into k classes based on a distance metric as similarity measure. Herein, the city block distance is used and it is given as following:

$${\text{D}}_{\text{City}-\text{block}}\left(M,N\right)=\sum_{J=0}^{n}\left|M_{J}-N_{J}\right|.$$ (13)

#### Decision Tree

Decision tree is a supervised machine learning technique for inducing a decision tree from training data. The decision tree predictive model will map the observations against an item to deduce the conclusion about its targeted value. Decision trees are grown using training data. Starting at the root node, the data is recursively split into subsets. In each step the best split is determined based on a criterion (Reinders et al., 2019). Commonly used criteria are G index and entropy index:

$$\text{G}\;\text{Index}:G\left(E\right)=1-\sum_{j=1}^{c}p_{j}^{2},$$ (14)

$$\text{Entropy}\;\text{index}\;\text{: }H\left(E\right)=-\sum_{j=1}^{c}p_{j}\log p_{j}.$$ (15)

The decision tree works on randomly chosen n training samples from the handwritten signature dataset. It creates a root and assigns the sample data to each node, this is repeated until all nodes comprise a single sample from same class, randomly selects m variables from M possible variables. Picks the best split feature and threshold the samples using the G index and entropy index and splits the node into two child nodes and pass the corresponding subsets.

#### Support Vector Machine

SVM is a supervised binary classifier. It works on a decision boundary in multidimensional feature vector space that is able to separate the classes of training set of vectors with known class labels. Geometrically, SVM are those training patterns that are closest to the decision boundary. SVM algorithm merely seeks out the separating hyperplane with the highest margin (Gornale. et al., 2016).

The proposed method is represented in the form of Algorithm as given below:

Input: Handwritten signature image.

Output: Writer’s gender classification.

Step 1: Input a handwritten signature image.
Step 2: Perform pre-processing of input image that includes noise removal and resizing by normalizing to a size of 150 × 150.
Step 3: Compute features
1) Local Binary Patterns, Histogram of Oriented Gradients and Statistical features individually.
2) Fusion of Local Binary Patterns, Histogram of Oriented Gradients and Statistical features.
Step 4: Classify the fused features using k-NN, decision tree and SVM classifiers and output the writer’s gender class.

End.

## Experimental Results and Analysis

The experimentation of the proposed method is carried out using own dataset which is described in *Dataset*. The dataset contains a total 4,790 signature images which are collected from 250 male and 229 female volunteers belonging to different age groups. Firstly, the performance of LBP, HOG and statistical features is investigated separately. With local binary patterns (LBP), 58 features are computed and then classified. By using HOG, 10,404 features are extracted and then classified. Next, 8 statistical features are extracted and then classified. In each case, k-NN, decision tree and SVM binary classifiers are employed.

Then, feature level fusion of Local Binary Patterns, Histogram of Oriented Gradients and Statistical and Textural features has been performed by concatenation of feature vectors. The fused features are stored as final feature vector. Finally, these features are classified by applying k-NN, decision tree and SVM binary classifiers.

To initiate the experiment, independent performance of LBP features of the handwritten signatures images is examined. It is observed that the highest accuracy of 96.11% is achieved by Decision Tree, and the lowest accuracy of 92.31% obtained by K-NN classifier. Similarly, in case of the independent analysis of HOG, it is found that the highest accuracy of 97.78% is achieved by Decision Tree, and the lowest accuracy of 94.55% obtained by K-NN classifier. Also, in case of the independent analysis of Statistical features, the highest accuracy of 92.65% is achieved by Decision Tree, and the lowest accuracy of 82.12% obtained by K-NN classifier.

Further, the fusion of different features were tested over the signature database and it was noted that, by fusing HOG and LBP features, the highest accuracy of 97.87% is achieved by Decision Tree, and the lowest accuracy of 94.53% obtained by K-NN classifier. Further, with the fusion of LBP and Statistical features, the highest accuracy of 96.20% is achieved by Decision Tree, and the lowest accuracy of 82.25% obtained by K-NN classifier. Also, in case of the fusion of HOG and the statistical features, the highest accuracy of 98.37% is achieved by Decision Tree, and the lowest accuracy of 81.67% obtained by K-NN classifier. Finally, the fusion of LBP, HOG, Statistical features yielded enhanced accuracy of 98.72% by Decision Tree, and 96.17% obtained by K-NN classifier. Table 1 contains the experimental results in terms of classification accuracy and confusion matrix, for each of the features and fusion of features, obtained by the binary classifiers, namely, k-NN, decision tree and SVM.

**TABLE 1:** Classification accuracy of the proposed method and confusion matrix, for each of the features and fusion of features, obtained by the binary classifiers, namely, k-NN, decision tree and SVM.

Features	k-NN Classifiers			Decision Tree Classifier			Support Vector Machine		
LBP	92.31	2092	170	96.11	2,191	87	100	2,290	00
		198	2,330		99	2,413		00	2,500
HOG	94.55	2,220	191	97.78	2,242	58	100	2,290	00
		70	2,309		48	2,442		00	2,500
Statistical	82.12	1846	412	91.44	2082	202	59.39	1,328	1883
		444	2088		208	2,298		962	617
HOG + LBP	94.53	2,219	191	97.87	2,250	62	100	2,290	00
		71	2,309		40	2,438		00	2,500
LBP + statistical	82.25	1847	407	96.20	2,209	101	63.50	1,143	601
		443	2093		81	2,399		1,147	1899
HOG + statistical	81.67	1819	407	98.37	2,252	40	100	2,290	00
		471	2093		38	2,460		00	2,500
LBP + HOG + statistical	95.69	2,240	156	98.68	2,270	43	100	2,290	00
		50	2,344		20	2,457		00	2,500
Textural	78.93	1753	472	86.17	1971	343	52.19	2,290	2,500
		537	2,208		319	2,157		00	00
LBP + Textural	58.91	1,302	1834	96.11	2,202	95	59.51	1,366	573
		988	666		88	2,405		924	1927
HOG + Textural	100	2,290	00	98.62	2,268	44	100	2,290	00
		00	2,500		22	2,456		00	2,500
Statistical + Textural	82.12	1846	412	92.65	2,102	164	60.79	1,119	707
		444	2088		188	2,336		1,171	1793
LBP + HOG + Textural	95.45	2,221	148	98.66	2,270	44	100	2,290	00
		69	2,352		20	2,456		00	2,500
LBP + HOG + Textural + statistical	96.17	2,255	148	98.72	2,269	40	100	2,290	00
		35	2,352		21	2,460		00	2,500

**FIGURE 5:**
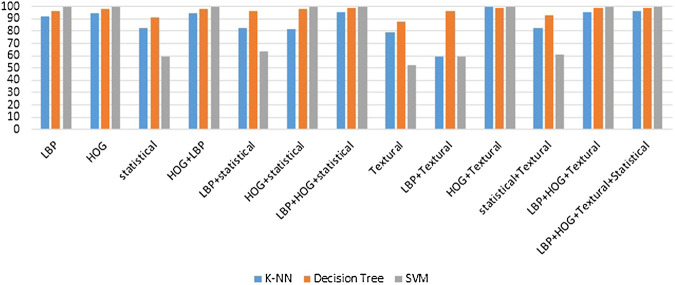
Graphical representation of features and classifiers.

The performance of the proposed algorithm is analyzed in terms of the metrics, namely, precision, F_Score, True Positive Rate (Recall) and False Positive Rate (Specificity), which are defined by Eqs. 16–19.

$$\text{Precision}\;=\;\frac{TP}{TP+FP},$$ (16)

$$\text{F\_Score}\;=\;\frac{2\;\text{*}\;\text{Precision}\;\text{*}\;\text{True Positive Rate}}{\text{Precsion}\;+\;\text{True Positive Rate}},$$ (17)

$$\text{True Positive Rate}\;=\;\frac{TP}{TP+FN},$$ (18)

$$\text{False Positive Rate}\;=\;\frac{FP}{FP+TN},$$ (19)

where, TN denotes True Negative, TP denotes True Positive, FP denotes False Positive and FN denotes False Negative. The performance comparison of the individual features with the fused feature is presented in Table 2 for each of the classifiers used in the classification experiments and also in Figure 5 graphically.

**TABLE 2:** The performance comparison of the individual features with the fused features is for each of the classifiers, namely, k-NN, Decision Tree and SVM.

	k-NN Classifier					Decision Tree					Support Vector Machine				
Features	Precision	F_score	TPR	Accuracy	FPR	Precision	F_Score	TPR	Accuracy	FPR	Precision	F_score	TPR	Accuracy	FPR
LBP	0.92	0.91	0.91	92.3	0.06	0.96	0.95	0.9	96.1	0.034	1	1	1	100	0
HOG	0.92	0.94	0.96	94.5	0.07	0.97	0.97	0.9	97.7	0.232	1	1	1	100	0
Statistical	0.81	0.81	0.80	82.1	0.16	0.91	0.90	0.9	91.4	0.080	0.58	0.59	0.5	59.39	0.75
LBP + HOG	0.92	0.94	0.96	94.5	0.07	0.97	0.97	0.9	97.8	0.024	0.50	0.34	0.2	52.19	0
LBP + statistical	0.81	0.81	0.81	82.2	0.16	0.95	0.96	0.9	96.2	0.080	1	1	1	100	0.24
HOG + statistical	0.81	0.80	0.79	81.6	0.16	0.98	0.98	0.9	98.3	0.024	0.62	0.63	0.6	63.05	0
LBP + HOG + Statistical	0.95	0.95	0.95	95.6	0.06	0.98	0.98	0.9	98.68	0.040	1	1	1	100	0
Textural	0.78	0.78	0.78	78.9	0.18	0.86	0.86	0.86	86.17	0.139	0.50	0.34	0.26	52.19	0
LBP + textural	0.58	0.58	0.59	58.9	0.73	0.96	0.96	0.9	96.11	0.017	0.58	0.59	0.6	59.51	0.22
HOG + textural	1	1	1	100	0	0.98	0.98	0.9	98.62	0.117	1	1	1	100	0
Statistical + textural	0.82	0.82	0.82	82.1	0.16	0.92	0.92	0.9	92.65	0.038	0.60	0.60	0.6	60.79	0.28
LBP + HOG + Textural	0.95	0.95	0.95	95.4	0.05	0.98	0.98	0.9	98.66	0.017	1	1	1	100	0
LBP + HOG + Statistical + Textural	0.96	0.96	0.96	96.1	0.05	0.98	0.98	0.9	98.72	0.016	1	1	1	100	0

### Comparative Analysis

To realize the effectiveness of the proposed method, it is compared with the similar works present in literature and are depicted in Table 3. A. A. M. Abushariah et al. (Abushariah et al., 2012) have performed signature based gender classification using the global features, namely, Height, width and Area on own database of 3,000 signatures of 50 males and 50 females. By using Artificial Neural Network (ANN), an average accuracy of 76.20 and 74.20% for male and female was obtained. Prasentjit Maji et al. (Maji et al., 2015) have performed gender classification based on offline handwritten signature by using basic statistical features over a dataset of 500 signatures. By using Back Propagation Neural Network, an accuracy of 84% was achieved. Danilo R.C. Bandeira et al. (Cavalcante Bandeira et al., 2019) have investigated the impact of the combination of a handwritten signature and keyboard keystroke dynamics for gender prediction. From 100 participants keystroke characteristics and handwritten images were collected. By extracting basic Static statistical and dynamic features obtained an average accuracy of 68.03% with Multi-Layer perceptron. Moumita Pal et al. (pal et al., 2018) have performed gender classification using Euler number based feature extraction on 500 Hindi handwritten signatures images. Based on Back Propagation Neural Network classification, an average accuracy of 88.80% is obtained. The drawback of the reported works is that the database of limited sizes are used for experiments. Whereas, the proposed method outperformed all these methods by using the fusion of LBP, HOG, Statistical and Texture features with decision tree classifier, wherein a relatively larger database consisting of 4,790 signature images is used. The proposed method based on feature fusion has yielded an accuracy of 98.72%, which is an encouraging result. Table 3 shows a summary of the related methods in the literature, features, classifiers and datasets used to achieve the reported classification accuracy. It is observed the proposed method based on feature fusion has yielded higher accuracy of gender classification than other methods.

**TABLE 3:** Summary of the related methods in the literature, features, classifiers and datasets used to achieve the reported classification accuracy.

Methods	Features	Database	Classifier	Result (%)
Abushariah et al. (2012)	Global features like height, width and area	3,000 signatures	Artificial neural network	76.20%
Maji et al. (2015)	Statistical features	500 signatures	Back propagation neural network	84%
R et al. (2019)	Static, statistical features	100 signature	Support vector machine	68.03%
Pal et al. (2018)	Statistical features with euler number	500 Hindi handwritten signatures	Support vector machine and back propagation neural network	80%
Proposed method	Local binary patterns, histogram of oriented gradient and statistical features	4,790 images	k-NN classifier	96.17%
			Decision tree	98.72%
			Support vector machine	100%

## Conclusion

In the present study, an effective method for gender identification based on handwritten signature is proposed using feature fusion and machine learning. Although from the related work, it is observed that researchers who have worked on gender classification using different methodologies and obtained some promising results with own datasets or standard datasets, there is still a large scope for developing a robust algorithm using effective discriminatory features. In the proposed method, feature-level fusion of LBP, HOG, Statistical and Textural features is experimented on own dataset comprising 4,790 signatures images (250 males and 229 females) of good quality. It is found that the proposed algorithm produces accuracies of 96.17% for k-NN, 98.72% for Decision Tree and 100% for Support Vector Machine, respectively. In future, the classification problem of gender determination from handwritten signatures and other biometrics using deep learning will be studied.

## Data Availability

The raw data supporting the conclusion of this article will be made available by the authors, without undue reservation.
